# Interleukin-5 Supports the Expansion of Fas Ligand-Expressing Killer B Cells that Induce Antigen-Specific Apoptosis of CD4^+^ T Cells and Secrete Interleukin-10

**DOI:** 10.1371/journal.pone.0070131

**Published:** 2013-08-05

**Authors:** Matthew W. Klinker, Tamra J. Reed, David A. Fox, Steven K. Lundy

**Affiliations:** 1 Graduate Program in Immunology, University of Michigan, Ann Arbor, Michigan, United States of America; 2 Division of Rheumatology, Department of Internal Medicine, University of Michigan, Ann Arbor, Michigan, United States of America; INSERM-Université Paris-Sud, France

## Abstract

Beyond their critical role in humoral immunity, B lymphocytes can employ a variety of immunomodulatory mechanisms including expression of the apoptosis-inducing molecule Fas ligand (FasL; CD178). Here, we extensively characterized the surface phenotype of FasL^+^ killer B cells, showing they are enriched in the IgM^high^CD5^+^CD1d^high^ B cell subset previously reported to contain a higher frequency of B cells producing interleukin-10 (IL-10). A rare population of B cells expressing IL-10 was present among FasL^+^ B cells, but most FasL^+^ B cells did not produce IL-10. We also identify interleukin-5 (IL-5) as a novel inducer of killer B cell function. Constitutively FasL^+^ B cells expressed higher levels of the IL-5 receptor, and treating B cells with IL-5 and CD40L resulted in the expansion of a B cell population enriched for FasL^+^ cells. B cells stimulated with IL-5 and CD40L were potent inducers of apoptosis in activated primary CD4^+^ T cells, and this killing function was antigen-specific and dependent upon FasL. IL-5 also enhanced IL-10 secretion in B cells stimulated with CD40L. Taken together these findings elucidate the relationship of FasL^+^ B cells and IL-10-producing B cells and demonstrate that IL-5 can induce or enhance both killer B cell activity and IL-10 secretion in B cells. Finally, we found that the killer B cell activity induced by IL-5 was completely blocked by IL-4, suggesting the existence of a previously unknown antagonistic relationship between these type-2 cytokines in modulating the activity of killer B cells. Targeting this IL-5/IL-4 signaling axis may therefore represent a novel area of drug discovery in inflammatory disorders.

## Introduction

B lymphocytes are best known as the mediators of humoral immunity, and in this capacity are crucial for host defense and maintaining homeostasis with commensal microbes. Despite their essential role as effector cells, there is also evidence for immunosuppressive “regulatory” B cells in several mouse models of human autoimmune diseases, including experimental autoimmune encephalomyelitis [1], [2], chronic intestinal inflammation [3], type 1 diabetes [4], [5], systemic lupus erythematosus [6], [7], and collagen-induced arthritis [8]. While B cell-mediated immunosuppression by secretion of the anti-inflammatory cytokine interleukin-10 (IL-10) has received much recent attention, there are several reports of suppressive effects of B cells independent of IL-10, including in mouse models of type 1 diabetes and multiple sclerosis [5], [9]–[11]. Additionally, it was recently shown that selective deletion of IL-10 in B cells did not affect disease parameters in a mouse model of lupus, suggesting that the *in vivo* effects of endogenous regulation by IL-10-producing B cells may be more subtle than previously thought [12]. Therefore, understanding the full repertoire of immunosuppressive mechanisms employed by B cells is crucial for appreciating their role in maintaining self-tolerance [13].

One alternative immunosuppressive mechanism used by B cells is the expression of death-inducing ligands such as Fas ligand (FasL; CD178). Upon binding the Fas receptor (CD95), FasL induces apoptosis in target cells such as activated peripheral CD4^+^ T cells [14]. Conceptually, FasL^+^ killer B cells uniquely possess the potential for suppression that is both *antigen specific*, as only CD4^+^ T cells recognizing antigens presented by FasL^+^ B cells are targeted, and *permanent*, as FasL signaling results in cell death rather than suppression [15]. Despite this, relatively little is known about FasL^+^ B cells in comparison to IL-10-producing B cells.

FasL^+^ B cells were reported to be induced by infection with *S. mansoni* and were enriched in the splenic CD5^+^ B cell subset [16], [17]. Activated B cells expressing FasL and TGF-β have also been reported to delay the onset of diabetes in non-obese diabetic (NOD) mice, and the frequency of FasL^+^ B cells is reduced in mice with severe autoimmune arthritis relative to those with mild or no arthritis [5], [18]. Bone marrow cells treated with the TLR-9 agonist CpG are enriched for B cells that express high levels of FasL and protect NOD mice from type 1 diabetes upon adoptive transfer [11]. Additionally, these CpG-elicited FasL^+^ B cells induced FasL-mediated apoptosis in CD4^+^ T cells *in vitro* and showed no evidence of increased IL-10 secretion. B cells from Fas-deficient MRL/*lpr* mice also overexpress FasL, and kill Fas-susceptible target cells with an efficiency similar to that of NK cells [19]. Mice with a B cell-specific loss of FasL spontaneously develop autoantibodies despite the fact that T cells in these animals are FasL-sufficient [20]. In a male-to-female skin graft model, transfer of B cells from wild-type males prior to skin grafting induced tolerance in female recipients, whereas FasL-deficient B cells were unable to transfer tolerance [21]. Taken together, these studies demonstrate that FasL^+^ B cells potentially play a role in the maintenance of peripheral tolerance and are elicited by diverse stimuli and inflammatory conditions.

This study focused on determining the relationship of FasL^+^ killer B cells to IL-10-producing B cells and identifying potential growth factors for these cells. We found that B cells were the predominant FasL-expressing lymphocyte population in the spleen, and while modestly enriched in the CD5^+^CD1d^high^ B cell subset displayed a heterogeneous surface phenotype. FasL^+^ B cells showed higher surface expression of the receptor for IL-5 than FasL^−^ B cells, and stimulating B cells with IL-5 and CD40L resulted in an expanded population that was enriched for FasL^+^ B cells and secreted IL-10. B cells stimulated with IL-5 and CD40L were exceptionally potent inducers of apoptosis in activated primary CD4^+^ T cells, and this killing activity was both antigen-specific and FasL-dependent. B cells treated with CD40L and IL-4, another type-2 cytokine, displayed no killing activity, even if treated in conjunction with IL-5. The results herein demonstrate that the combined stimulus of IL-5 and CD40L *in vitro* results in an expanded population of B cells capable of both inducing FasL-mediated apoptosis of CD4^+^ T cells and secreting IL-10. These results imply that IL-5 receptor signaling is a potential target for therapeutic intervention in patients with autoimmune diseases, and provide evidence for a novel antagonistic relationship between IL-4 and IL-5 in the function of FasL^+^ killer B cells.

## Results

### Frequency and Surface Phenotype of FasL^+^ B Cells

**Figure 1:**
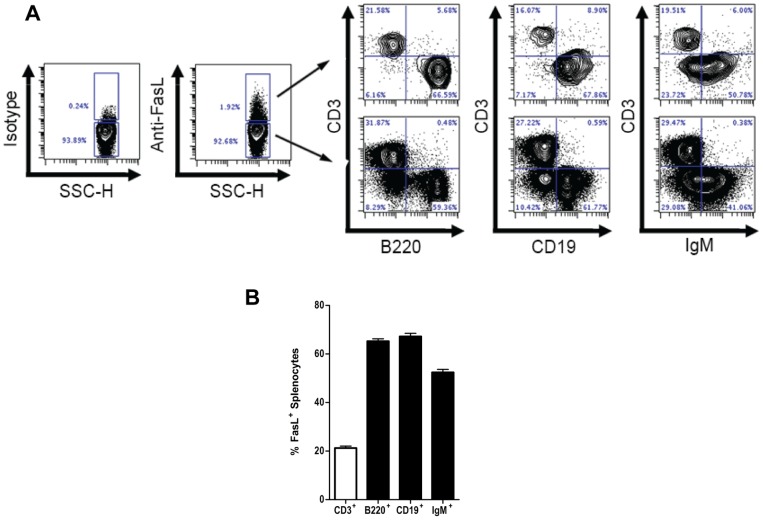
The majority of FasL^+^ splenocytes are B cells. Splenocytes from naïve mice were stained for surface expression of FasL and the cellular composition of FasL^−^ and FasL^+^ subsets was examined by flow cytometry. (**A**) Representative dot plots of splenocytes stained with a T cell marker (CD3) and three independent B cell markers (B220, CD19, and IgM). (**B**) The frequency (mean ± SEM) of cells staining positive for the markers presented in (A) among FasL^+^ splenocytes. Data are representative of more than 5 independent experiments with at least three animals per experiment.

To measure the frequency of B cells among all FasL^+^ splenocytes, we compared the expression of B cell-specific surface markers among FasL^+^ and FasL^−^ splenocytes in naïve DBA/1 mice. As determined by surface expression of B220, CD19, and IgM, we found that B cells comprised the majority (∼60%) of constitutively FasL^+^ splenocytes (Figures 1A and 1B). As expected, CD3^+^ T cells accounted for most of the remaining FasL^+^ splenocytes. Similar frequencies of FasL^+^ B cells were found in C57BL/6 and CBA/J mice housed under identical conditions (data not shown). These data demonstrate that B cells represent the major source of cells expressing surface FasL in the mouse spleen.

**Figure 2:**
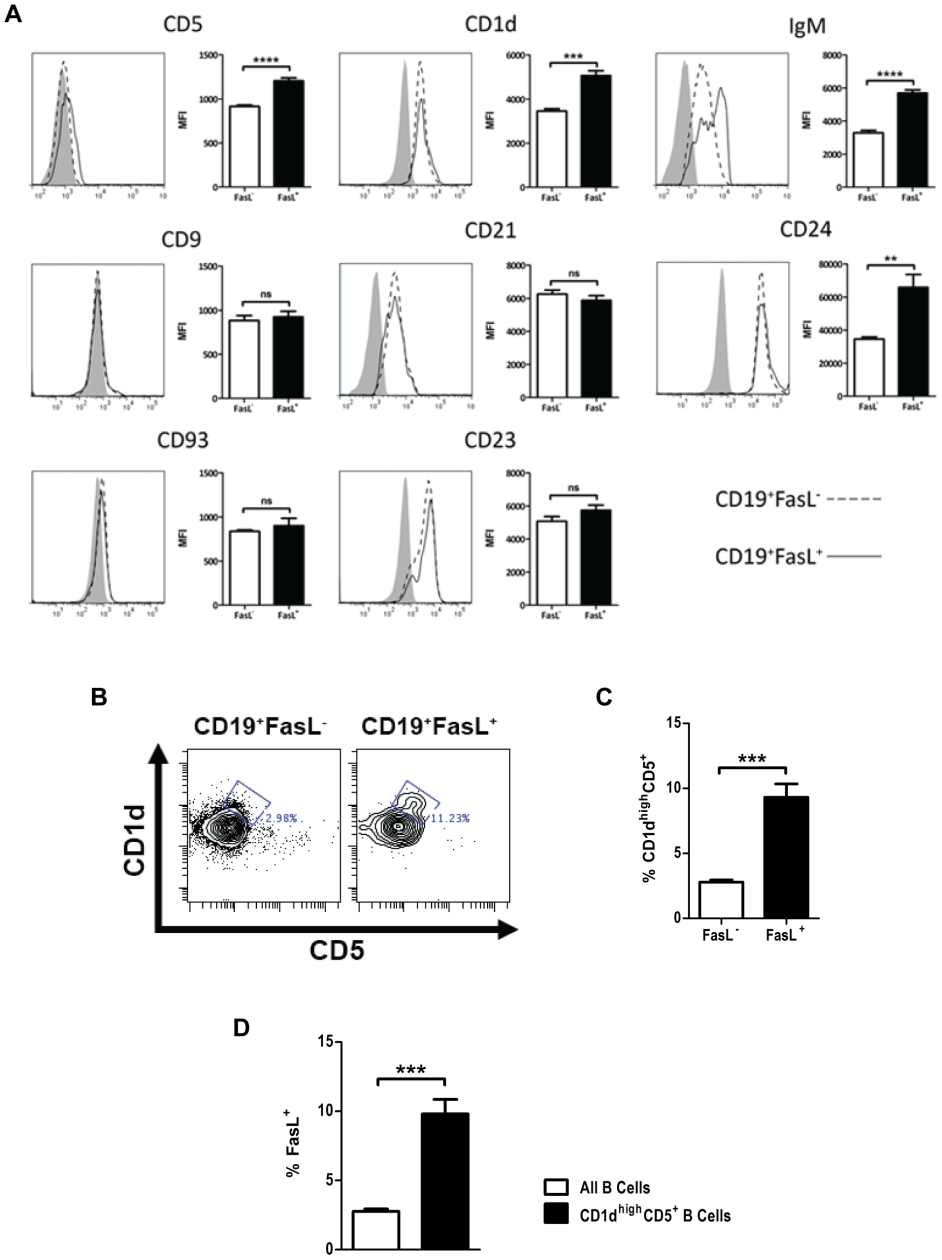
FasL^+^ B cells are enriched in the CD5^+^CD1d^high^ subset. (**A**) Representative histograms showing relative surface expression of markers for B cell subsets in CD19^+^FasL^−^ and CD19^+^FasL^+^ B cells. Bar graphs are the average median fluorescence intensity from replicate animals. Grey histograms represent isotype control antibody staining. (**B**) FasL^−^ and FasL^+^ B cells were stained for co-expression of CD5 and CD1d. (**C**) The frequency of CD5^+^CD1d^high^ cells (mean ± SEM) among FasL^−^ and FasL^+^ B cells in replicate animals was measured. (**D**) Reciprocally, the frequency of FasL^+^ cells among all B cells and CD5^+^CD1d^high^ B cells was measured. Data are representative of four independent experiments using at least 3 animals per experiment. **p<0.01, ***p<0.001, and ****p<0.0001.

B cells capable of secreting IL-10 are reportedly enriched in several B cell subsets. In particular, IL-10-producing B cells are enriched in both the CD5^+^CD1d^high^ (sometimes referred to as “B10” cells) and CD23^+^CD21^high^IgM^high^ transitional 2-marginal zone precursor (T2-MZP) B cell subsets, although most IL-10-secreting B cells are distributed among other B cell subsets [22]–[24]. Previous studies in our laboratory had identified that FasL^+^ B cells were primarily found in the lung and splenic CD5^+^ B cell subsets but had not been characterized further [17], [18]. To understand how FasL^+^ B cells relate to previously-identified B cell subsets, we compared surface expression of several B cell subset markers in FasL^+^ and FasL^−^ B cells. We found that CD19^+^FasL^+^ B cells displayed a modest but statistically significant enhancement in surface expression of CD5, CD1d, IgM, and CD24 than CD19^+^FasL^−^ B cells (Figure 2A). Surface staining for CD9, CD93, CD21 and CD23 was neither higher nor lower than levels in FasL^−^ B cells. Co-staining for CD5 and CD1d showed that CD5^+^CD1d^high^ B cells were more frequent among FasL^+^ B cells than FasL^−^ B cells (Figures 2B and 2C). Reciprocally, the CD5^+^CD1d^high^ B cell subset had a nearly four-fold enrichment for FasL^+^ B cells (Figure 2D). We did not find evidence of an increased frequency of CD23^+^CD21^high^ T2-MZP B cells among the constitutively FasL^+^ B cells than in FasL^−^ B cells (Figure S1). Thus, while FasL^+^ B cells were enriched in the same subset reported to contain B10 cells, FasL^+^ B cells were not exclusively CD5^+^CD1d^high^, indicating that FasL^+^ B cells, like IL-10-producing B cells, are not limited to a single subset.

**Figure 3:**
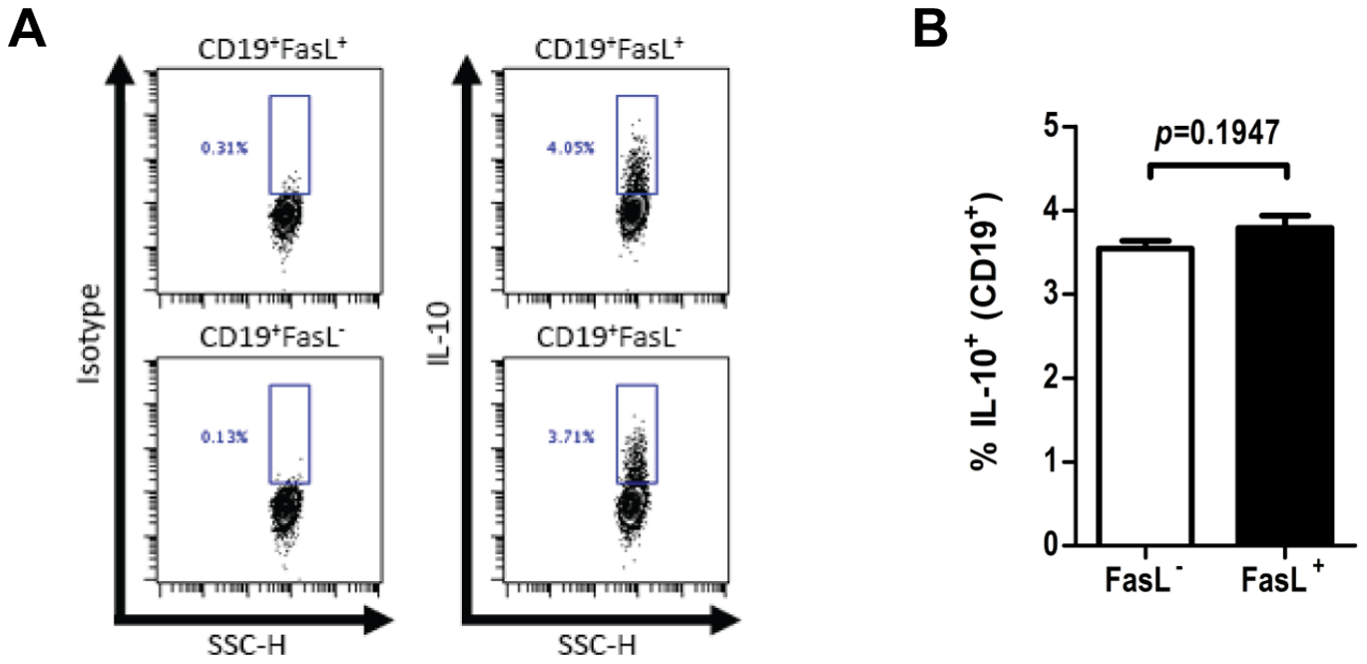
IL-10-producing B cells are not enriched in the FasL^+^ subset. FasL^−^ and FasL^+^ splenic B cells from naïve mice were sorted by FACS and stimulated with PMA, ionomycin, and LPS in the presence of monensin for five hours. (**A**) Following stimulation, FasL^−^ and FasL^+^ B cells were fixed and stained for intracellular IL-10. (**B**) Frequency of IL-10-producing B cells (mean ± SEM) among CD19^+^FasL^−^ and CD19^+^FasL^+^ subsets.

Given that FasL^+^ B cells shared at least some phenotypic similarities with IL-10-producing B cells, we sought to determine whether these were discrete or overlapping cell populations. We initially attempted to stain for both FasL and IL-10 in the same cells using established protocols [25], but culture conditions required to detect intracellular IL-10 eliminated surface FasL staining (Figure S2). As an alternative, we sorted B cells from naïve mice into CD19^+^FasL^−^ and CD19^+^FasL^+^ populations by FACS. Sorted B cells were then cultured for 5 hours with PMA, ionomycin, and LPS in the presence of monensin, and subsequently fixed and stained for intracellular IL-10. We found that IL-10-producing B cells were present at similar frequencies among both FasL^+^ and FasL^−^ B cells (Figures 3A and 3B). While the majority of FasL^+^ B cells do not produce detectable IL-10 under these conditions, ∼4% of FasL^+^ B cells produced IL-10 upon *ex vivo* stimulation, suggesting that FasL^+^IL-10^+^B cells are extremely rare in naïve mice (<0.1% of all splenic B cells).

### IL-5 and CD40L Stimulation Expands a Population of B Cells Enriched for FasL Expression

**Figure 4:**
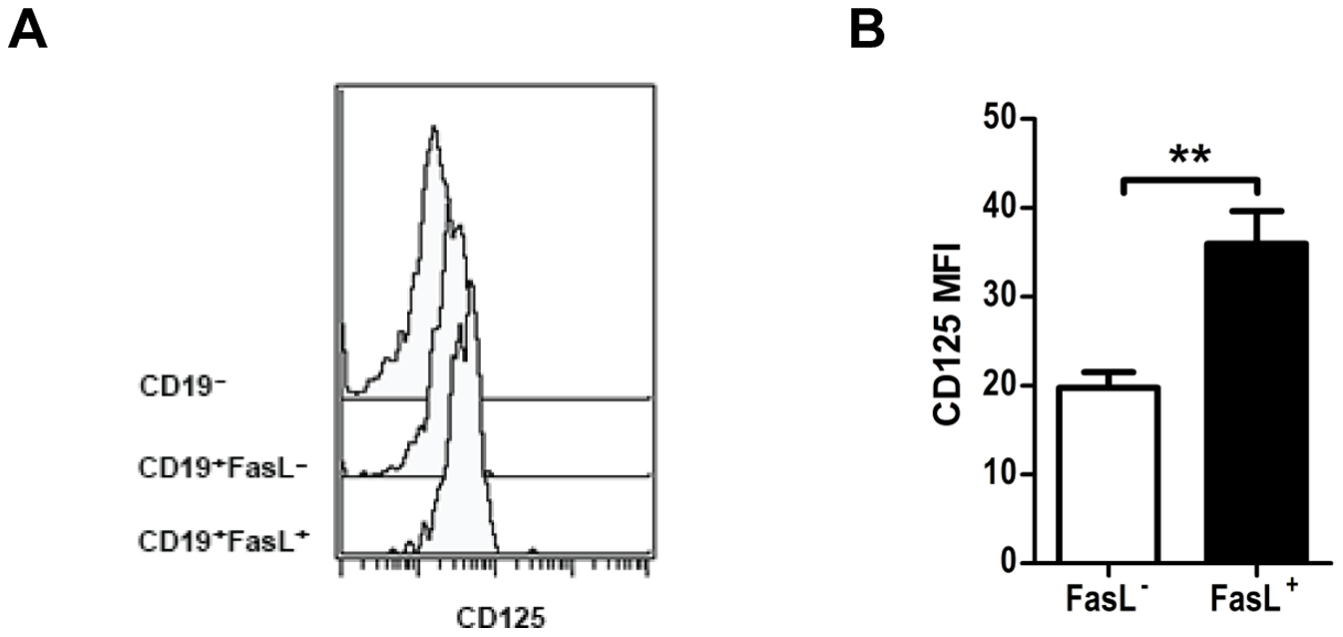
FasL^+^ B cells express higher levels of the IL-5 receptor than FasL ^−^ **B cells.** (**A**) Histograms comparing surface expression of CD125 in CD19^−^, CD19^+^FasL^−^ and CD19^+^FasL^+^ cells. (**B**) Quantification of median fluorescence intensity of CD125 expression in CD19^+^FasL^−^ and CD19^+^FasL^+^ B cells in replicate animals (mean ± SEM). **p<0.01.

As both FasL^+^ and IL-10-producing B cells are enriched in the CD5^+^CD1d^high^ B cells subset, we compared global gene expression between FACS-sorted CD5^+^CD1d^high^ and CD5^−^CD1d^high^ B cells by microarray to identify putative surface markers or growth factors for killer and/or regulatory B cells. These complete data and a full description of the methods used to obtain the cell populations compared are freely available from the NCBI GEO depository (Accession number GSE46245). We found that both the alpha (*Il5ra*) and beta (*Csf2rb*) chains of the IL-5 receptor were among the most differentially-expressed genes, with both genes expressed at greater than three-fold higher levels in CD5^+^CD1d^high^ B cells (data not shown). We therefore assessed surface expression of the alpha chain of the IL-5R (CD125) on FasL^+^ and FasL^−^ B cells by flow cytometry and observed that FasL^+^ B cells displayed higher CD125 expression than FasL^−^ B cells (Figures 4A and 4B).

**Figure 5:**
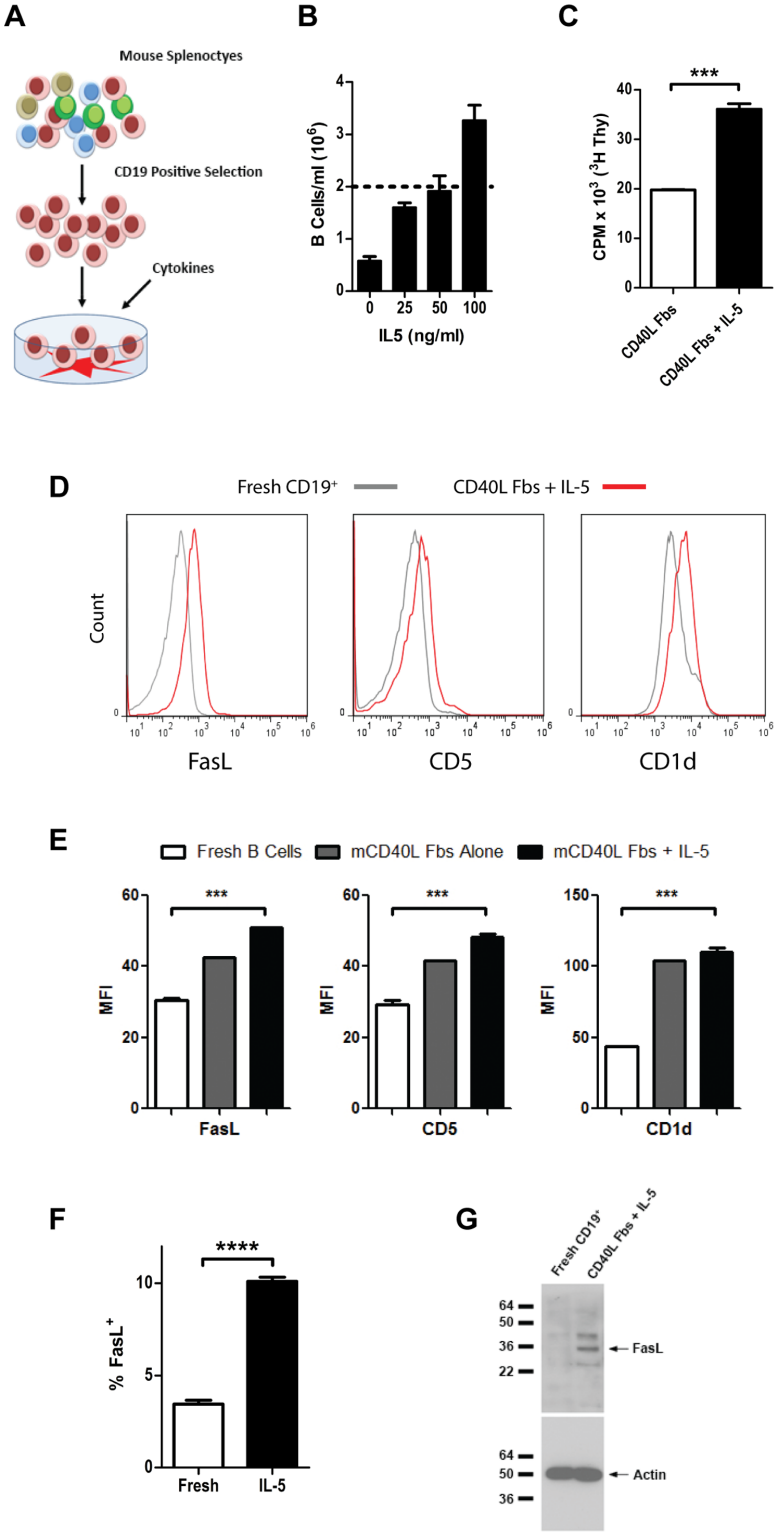
IL-5 and CD40L stimulation expands a population of B cells enriched for FasL expression. (**A**) Diagram of B cell co-culture experiments with CD40L-expressing fibroblasts for study of the effects of IL-5 on B cell growth and function. (**B**) CD19^+^ B cells were cultured as illustrated in (A) and harvested after five days. Viable B cells recovered from culture were quantified from replicate samples using a hemocytometer and trypan blue exclusion. The dotted line represents the concentration of B cells at the beginning of the experiment. (**C**) Proliferation of B cells cultured with CD40L-expressing fibroblasts in the presence or absence of IL-5 was assessed by the incorporation of ^3^H-thymidine. (**D**) B cells stimulated with CD40L in the presence or absence of IL-5 were stained for FasL, CD5, and CD1d and compared with freshly-isolated CD19^+^ B cells. (**E**) Median fluorescence intensity of surface markers stained as in (D) on freshly-isolated CD19^+^ B cells, B cells stimulated with CD40L, and B cells stimulated with CD40L and IL-5. (**F**) The frequency of FasL^+^ B cells among freshly-isolated CD19^+^ B cells and B cells stimulated with CD40L and IL-5 was measured by flow cytometry as in (D). (**G**) Cell lysates from equal numbers of freshly-isolated CD19^+^ B cells and IL-5-stimulated B cells were probed by immunoblot for FasL and β-Actin. Data are representative of more than four independent experiments. ***p<0.001.

Given that FasL^+^ B cells showed higher surface expression of the IL-5 receptor, we hypothesized that IL-5 could be a survival and/or growth factor for these cells. As B cell survival under most *in vitro* conditions is low, we adapted a technique used for the maintenance of human B cells for cultures of murine B cells. As outlined in Figure 5A, CD19^+^ B cells were positively selected from naïve mice by MACS and cultured with irradiated NIH-3T3 fibroblasts stably expressing a vector encoding murine CD40 ligand (CD40L Fb). Culturing CD19^+^ B cells on CD40L Fb allowed for maintenance of B cell cultures for up to 14 days and was dependent upon CD40L expression, as non-transduced fibroblasts did not increase B cell survival above that of B cells cultured in medium alone (data not shown). Using this culture system, we investigated the effects of adding recombinant murine IL-5 to cultures of CD19^+^ B cells. As seen in Figure 5B, the addition of IL-5 to CD19^+^ B cells cultured with CD40L Fb led to a dose-dependent increase in the number of B cells recovered after five days. At high concentrations of IL-5 (100 ng/mL), more B cells were collected after five days in culture than had been added, demonstrating that IL-5-induced proliferation of B cells. We confirmed increased proliferation in B cells treated with IL-5 by assessing ^3^H-thymidine incorporation after 5 days in culture (Figure 5C). Therefore, IL-5 promotes the growth and proliferation of B cells stimulated with CD40L.

Next, we compared B cells cultured with IL-5 and CD40L Fb to freshly-isolated CD19^+^ B cells for surface expression of FasL and markers associated with FasL^+^ B cells *in vivo*. B cells cultured with IL-5 and CD40L Fb showed higher surface expression of FasL, CD5, and CD1d (Figure 5D and 5E), and were significantly enriched for FasL^+^ B cells (Figure 5F). Immunoblotting for FasL confirmed this result, as IL-5-stimulated B cells had readily-detectable FasL protein whereas FasL was not detectable in an equivalent number of freshly-isolated CD19^+^ B cells (Figure 5G). Interestingly, the small residual population of living B cells remaining after five days of culture with CD40L Fb alone also displayed increased surface expression of FasL, CD5, and CD1d relative to freshly-isolated B cells (Figure 5E).

### B Cells Stimulated with IL-5 and CD40L Induce Antigen-specific Apoptosis in CD4^+^ T Cells

**Figure 6:**
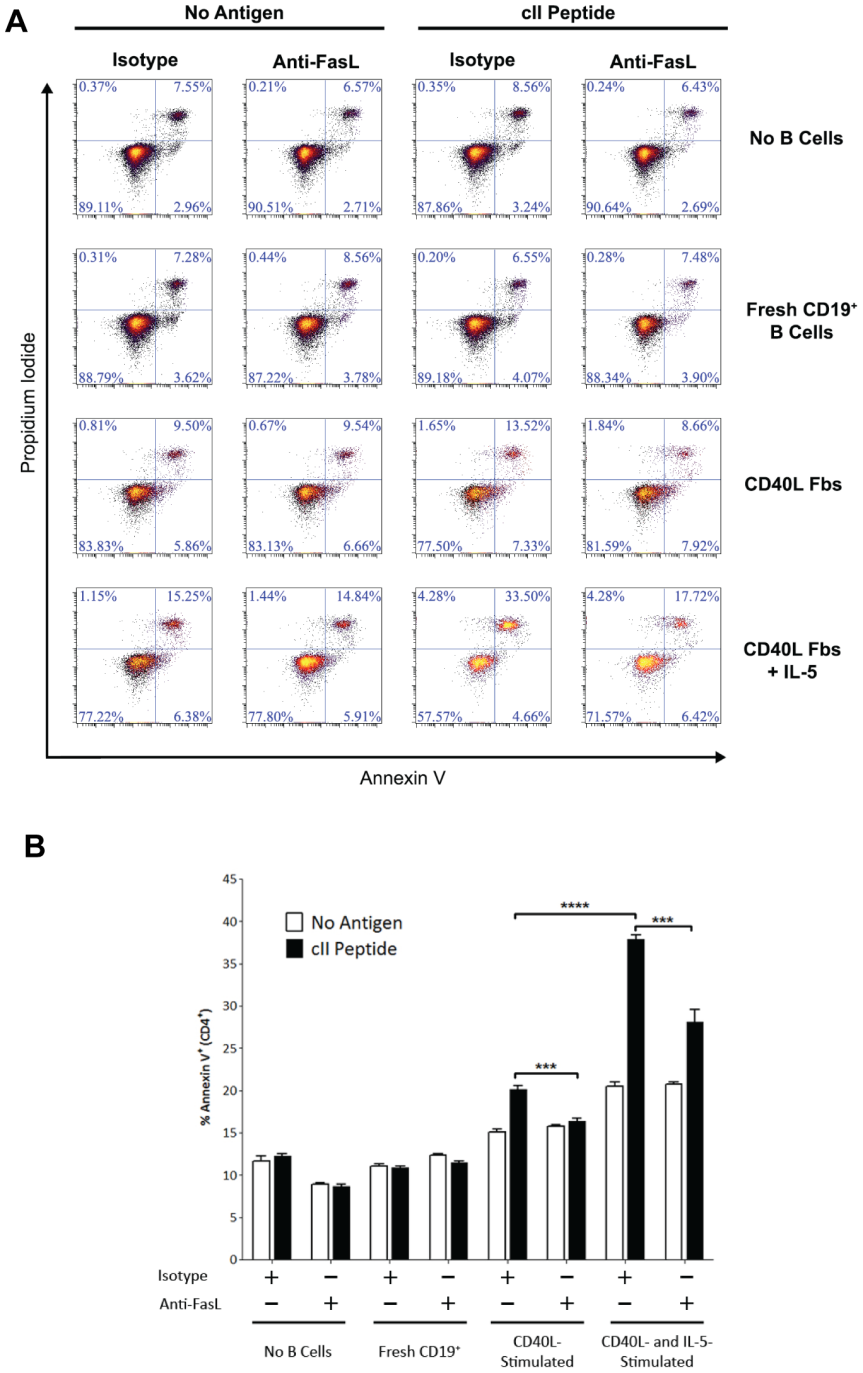
B cells stimulated with IL-5 and CD40L induce antigen-specific apoptosis in CD4^+^ T cells. Splenocytes were isolated from naive cII-TCR transgenic mice and stimulated *in vitro* with Concanavalin A. After three days in culture, CD4^+^ T cells were negatively-selected by MACS and co-cultured with freshly-isolated CD19^+^ splenic B cells or stimulated B cells at a 1∶1 ratio. B cells were incubated with anti-FasL blocking antibody or an isotype control antibody for 1 hour and washed with PBS prior to co-culture with CD4^+^ T cells. In wells with no B cells, anti-FasL antibody or isotype control antibody were directly added to culture wells to assess FasL-mediated “fratricide” between CD4^+^ T cells. Apoptosis was measured at 18 hours by flow cytometry as the percentage of CD4^+^cells that were positive for Annexin V staining. (**A**) Representative plots of gated CD4^+^ T cells for Annexin V and propidium iodide following co-culture with B cells. (**B**) Quantification (mean ± SEM) from a single experiment as described in (A). Data are representative of four independent experiments. ***p<0.001 and ****p<0.0001.

Naïve CD4^+^ T cells are relatively protected from FasL-mediated killing, but will readily undergo apoptosis in response to Fas signaling after activation. We therefore used *in vitro* activated CD4^+^ T cells from TCR transgenic mice as targets to test the apoptosis-inducing capacity of B cells. Splenocytes from cII-TCR transgenic mice were collected and cultured with Concanavalin A. After three days, activated CD4^+^ T cells were then negatively-selected by MACS and co-cultured at a 1∶1 ratio with B cells in the presence or absence of cognate peptide. After 18 hours, we assessed apoptosis among CD4^+^ T cells by Annexin V/propidium iodide staining (Figures 6A and 6B).

In the absence of cognate peptide, B cells stimulated with CD40L alone or in conjunction with IL-5 induced slightly higher levels of apoptosis than were induced by fresh CD19^+^ B cells (Figures 6A and 6B). In the presence of cognate peptide, however, the IL-5-stimulated B cells induced significantly higher levels of apoptosis in CD4^+^ T cells than either freshly-isolated CD19^+^ B cells or B cells stimulated with CD40L Fbs alone (Figures 6A and 6B). To test the role of B cell-derived FasL in this killing activity, we pre-treated B cells for one hour with an anti-FasL blocking antibody or isotype control antibody prior to co-culture with CD4^+^ T cells. Blocking FasL on B cells significantly abrogated the antigen-specific killing function of CD40L -stimulated and IL-5/CD40L-stimulated B cells (Figures 6A and 6B). Taken together, these results demonstrate that these stimulated B cells induce apoptosis in CD4^+^ T cells at a low effector-to-target ratio, and that this effect is antigen specific and FasL-dependent. B cells stimulated with CD40L Fb alone also induced antigen-specific apoptosis in T cells, but had significantly weaker killing activity relative to that of B cells stimulated with IL-5 and CD40L (Figure 6A and 6B).

### IL-5 Enhances Secretion of IL-10 in CD40L-stimulated B Cells

**Figure 7:**
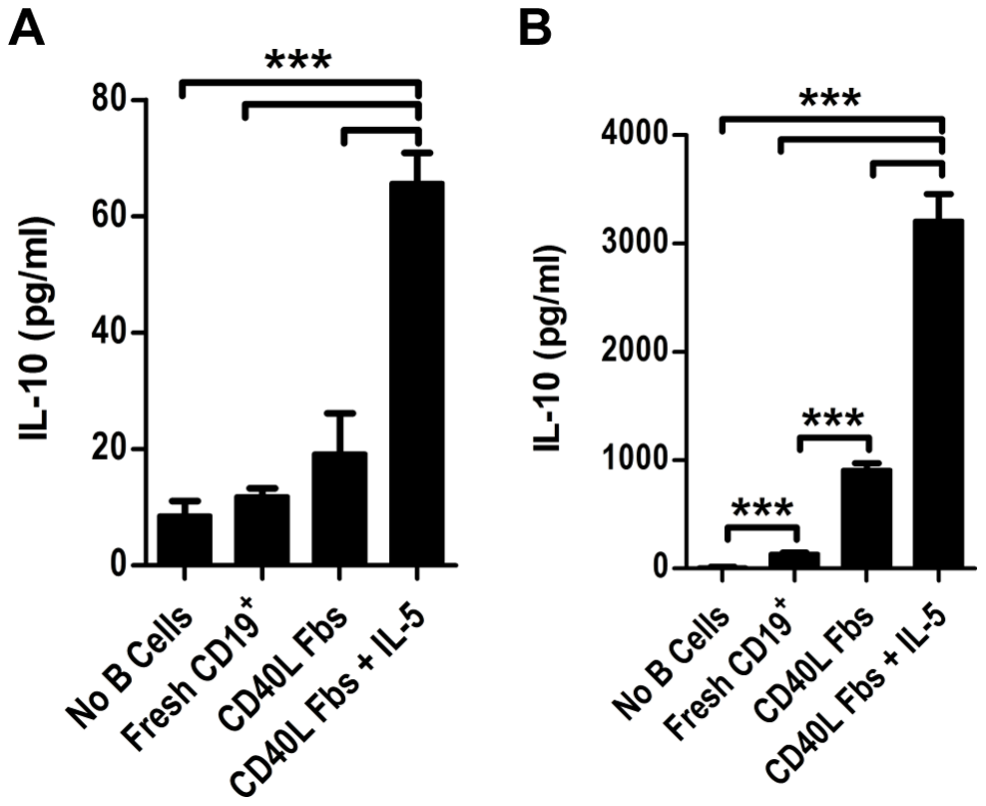
IL-5 enhances IL-10 secretion from CD40L-stimulated B cells. CD19^+^ B cells were isolated by MACS from naïve mice and cultured for five days with CD40L-expressing fibroblasts in the presence or absence of IL-5. B cells were then harvested, washed in PBS, and cultured overnight at equivalent cell concentrations (7.5×10^5^ viable cells/mL) in the presence or absence of stimulation with PMA and ionomycin. IL-10 in the culture supernatants was measured by ELISA. (**A**) IL-10 secretion from B cells after overnight culture with no further stimulation. (**B**) IL-10 secretion from B cells after overnight culture with PMA and ionomycin. Data are representative of at least three independent experiments (mean ± SEM). ***p<0.001.

Our initial microarray findings showed that expression of the IL-5 receptor was high in CD5^+^CD1d^high^ B cells, a population which is enriched for both FasL^+^ B cells and IL-10-producing B cells. We therefore sought to determine whether IL-5 also affected IL-10 secretion. Using the same system outlined in Figure 5A, we cultured B cells with CD40L Fb in the presence or absence of IL-5 for five days. Cultured B cells were then harvested, washed, and re-cultured at equal cell concentrations along with freshly-isolated CD19^+^ B cells. After 24 hours, culture supernatants were collected and IL-10 secretion was measured by ELISA. As seen in Figure 7A, B cells stimulated with IL-5 and CD40L produced detectable amounts of IL-10 without further re-stimulation, whereas B cells stimulated by CD40L alone or freshly-isolated B cells did not produce detectable IL-10. In response to stimulation with PMA and ionomycin, B cells stimulated with CD40L alone secreted IL-10, but this secretion was greatly enhanced by the addition of IL-5 (Figure 7B). From these data, we conclude that stimulation with IL-5 and CD40L, in addition to its ability to induce FasL-mediated killer function in B cells, also enhances IL-10 secretion.

### IL-5-mediated Induction of Killer B Cell Function is Inhibited by IL-4

**Figure 8:**
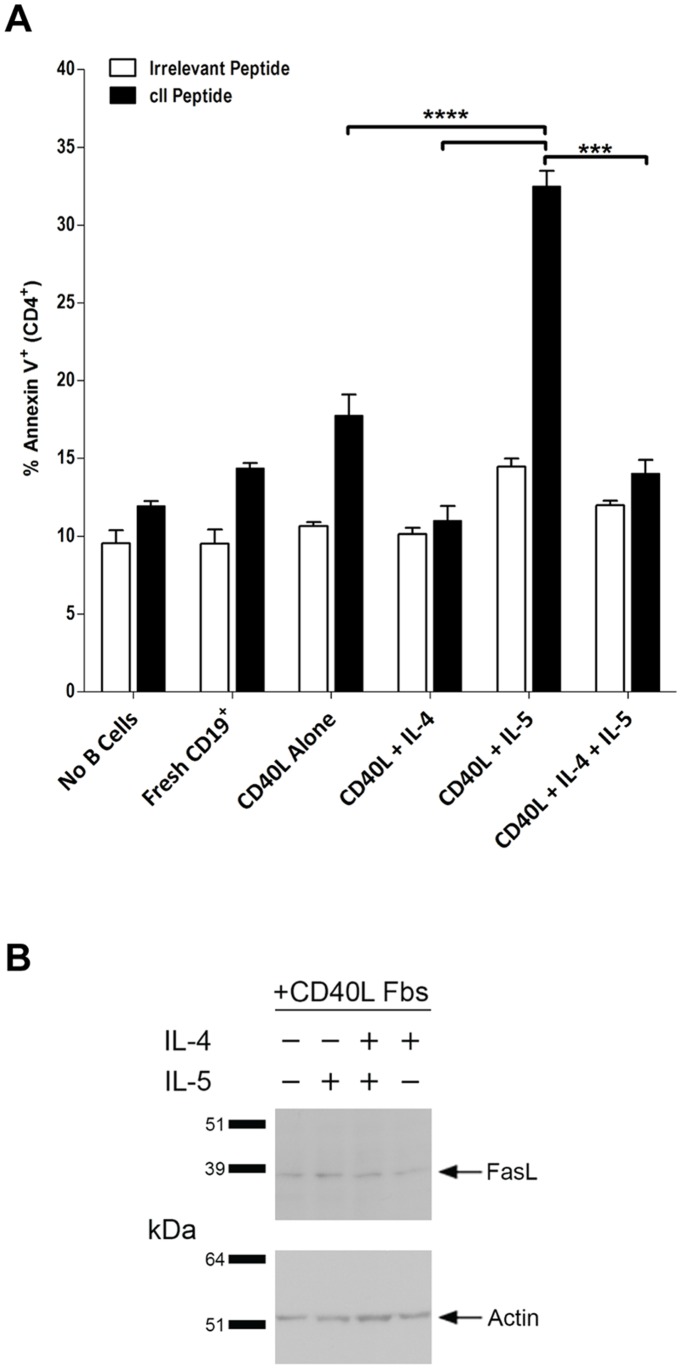
IL-5-mediated induction of killer B cell function, but not FasL expression, is inhibited by IL-4. CD19^+^ B cells from naïve mice were cultured for five days with CD40L Fb and IL-4, IL-5, or both cytokines concurrently. (**A**) The ability of B cells stimulated under these conditions to induce apoptosis in activated CD4^+^ T cells was then assessed as in Figure 6. Apoptotic cells were identified and enumerated (mean ± SEM) on the basis of positive staining for Annexin V by flow cytometry. (**B**) B cells were cultured with CD40L-expressing fibroblasts in the presence or absence of IL-4 and IL-5, or in the presence of both cytokines. Cells were harvested after 5 days in culture and cell lysates were probed for FasL and β-Actin proteins as in Figure 5G. ***p<0.001 and ****p<0.0001.

As IL-5 is associated with the type-2 immune response, we hypothesized that IL-4, the hallmark cytokine of the type-2 response, might have an enhancing effect on the IL-5-mediated killer B cell program. To test this, we assessed the apoptosis-inducing capacity of B cells cultured with CD40L Fb and IL-4, IL-5, or both cytokines simultaneously as in Figure 5A. B cells cultured with IL-4 and CD40L Fb did not display any detectable killing activity, in contrast to the measurable antigen-specific apoptosis induced by CD40L stimulation alone and the robust killing displayed by B cells stimulated with IL-5 and CD40L Fb. (Figure 8A). When both IL-4 and IL-5 were present in B cell cultures, the resulting cells again displayed no detectable killing activity (Figure 8A). These data therefore suggest that, rather than enhancing the effects of IL-5, IL-4 instead strongly inhibited the killing function induced by IL-5 in B cells.

Given its ability to dominantly inhibit the increased killing activity induced by IL-5, we hypothesized that IL-4 accomplished this inhibition by reducing FasL expression. Somewhat unexpectedly, we found essentially equivalent amounts of FasL protein (on a per cell basis) in all B cells stimulated with CD40L, regardless of the addition of cytokines (Figure 8B). These data suggest that the mechanism through which IL-4 inhibits IL-5-induced killer B cell function does not rely on reducing levels of FasL protein in B cells.

## Discussion

B cells can modulate an immune response through a variety of mechanisms including the expression of FasL [13], [21], [26]. The Fas/FasL signaling axis is critical for the maintenance of self-tolerance, as mice bearing natural mutations in genes coding for these molecules develop spontaneous systemic autoimmunity [27], [28]. We show here that B cells make up the majority of FasL-expressing splenocytes in naïve mice, suggesting that FasL^+^ B cells may play a role in maintaining immune homeostasis. FasL^+^ APCs have demonstrated efficacy in establishing tolerance upon adoptive transfer, as both endogenous unaltered B cells and APCs transduced with FasL-expression vectors mediate the induction of antigen-specific tolerance [21], [29], [30]. In light of these studies, further examination of endogenous FasL^+^ APCs is warranted to understand their importance in maintaining immune homeostasis and self-tolerance.

Our results demonstrate that FasL^+^ B cells have a surface phenotype that bears some similarity to that reported for the B10 subset of IL-10-producing B cells [22]. Specifically, FasL^+^ B cells are moderately enriched in the CD5^+^CD1d^high^ B cell subset, but only a minority of FasL^+^ B cells is found within this B cell subset. IL-10-producing B cells are also enriched among CD5^+^CD1d^high^ B cells, but only ∼25% of IL-10-producing B cells reside in the CD5^+^CD1d^high^ subset [22], [24]. Despite their shared enrichment in the CD5^+^CD1d^high^ B cell subset, IL-10-producing cells are not more frequent among FasL^+^ B cells than FasL^−^ B cells. The FasL^+^ B cells that do secrete IL-10, however, are extremely rare, making up<0.1% of all splenic B cells (∼2% of B cells are FasL^+^, and ∼4% of FasL^+^ B cells produce IL-10 after *ex vivo* stimulation). Although FasL^+^IL-10^+^ B cells might be more frequent in animals mounting an active immune response, their extreme paucity in naïve mice has made characterizing these cells further technically difficult.

FasL^+^ B cells also share some phenotypic characteristics with B-1a cells (CD5^+^IgM^high^) and marginal zone B cells (CD1d^high^). These innate-like B cell subsets share functional similarities, and arise from a common precursor population distinct from that of follicular B-2 cells [31]–[34]. Innate-like B cells secrete natural IgM antibodies that play a role in the elimination of apoptotic cells [35]–[37]. Natural antibodies in this scenario are anti-inflammatory as they aid in the clearance of apoptotic cells, and even in the absence of relevant antigens can directly inhibit the activation of antigen-presenting cells. Currently, only circumstantial evidence supports a link between killer B cells and innate-like B cells, and more study is required to determine the relationship between these cell types.

We also report here for the first time that that stimulating B cells with IL-5 in conjunction with CD40L results in an expanded B cell population enriched for FasL expression and able to secrete IL-10. This finding is consistent with our previous studies demonstrating that FasL^+^ B cells were increased in mice infected with *S. mansoni* and in a mouse model of chronic allergen-induced asthma, two conditions that result in an increase in T cell activation and production of IL-5 and other type 2 cytokines [17], [38]. It is important to note that the effects of IL-5 on the immune system appear to be pleiotropic, as both pro- and anti-inflammatory effects have been reported. IL-5 has a role in generating optimal antibody responses, as mice in which B cells are unable to respond to IL-5 signaling are defective in plasma cell differentiation and have reduced levels of circulating antibodies [39]. Accordingly, over-expression of IL-5 results in increased antibody levels and hypereosinophilia, and mice deficient in IL-5 receptor signaling have reduced levels of antibodies [40], [41]. In contrast, IL-5 is a critical survival factor for peritoneal B-1 cells, and administration of IL-5 leads to an increase in secretion of anti-inflammatory natural antibodies by B-1 cells [42], [43]. In a rat model of multiple sclerosis, IL-5 administration had therapeutic effects, acting at least in part by inducing FoxP3^+^ regulatory T cells [44]. Further work is needed in examining the role of IL-5 in autoimmunity, however, as mice deficient in IL-5 appeared to have a similar clinical course of EAE [45]. None-the-less, because of its ability to induce cytotoxic activity and increase production of both IL-10 and natural antibodies in B cells, the IL-5/IL-5 receptor axis makes for an intriguing target for therapeutic intervention in pathogenic inflammatory conditions.

While the *in vivo* targets of FasL^+^ B cells remain to be determined, we show here that IL-5 stimulated FasL^+^ B cells induce apoptosis in activated CD4^+^ T cells. This killing function was greatly increased upon the addition of antigen, suggesting that FasL^+^ B cells preferentially target T cells based upon antigen specificity. The potency of this killing effect is especially noteworthy, as an effector-to-target ratio 10–20 fold lower than usually required to measure cytotoxic activity resulted in significant levels of apoptosis in CD4^+^ T cells. While thymic negative selection of T cells is thought to eliminate those which respond to self-antigens, some autoreactive T cells are present among peripheral T cells [46]. As innate-like B cells frequently possess self-reactive BCRs, the antigens presented by FasL^+^ B cells would be expected to be enriched for self-antigens due to BCR-mediated endocytosis and processing of host-derived molecules [31]. FasL^+^ B cells may therefore play a role in maintaining self-tolerance by eliminating CD4^+^ T cells recognizing self-antigens that escape negative selection in the thymus. While it has been reported that mice with a targeted deletion of the *Fasl* gene in B cells show symptoms of autoimmunity, further work is needed to evaluate the importance of this mechanism in animal models of autoimmunity [20].

We also found that IL-4 dominantly inhibited the IL-5-induced killer function of B cells. Intriguingly, despite the vast difference in their killing activity there is no consistent difference in FasL protein levels (on a per cell basis) between B cells stimulated with IL-5 and CD40L and those stimulated with IL-4, IL-5, and CD40L (Figure 8B). Thus, IL-4 must inhibit the FasL-mediated killing function induced by IL-5 through other mechanisms. These data are in agreement with previous findings regarding induction of FasL^+^ B cells by IL-4 and IL-10 in the schistosome granuloma model [17]. While perhaps unexpected, this result is consistent with what is known regarding the complex and unusual regulation of FasL. Killer cells such as cytotoxic T cells and NK cells keep most FasL protein sequestered in the secretory lysosome, an endosomal-like intracellular compartment [41], [47]. FasL is then moved to the cell surface in response to signals received upon encounter with an appropriate target cell. There are several nodes of regulation in this process upon which IL-4 may act, such as preventing the phosphorylation events required for the transport of FasL to the secretory lysosome, inhibiting the movement of the secretory lysosome to the cell surface, or reducing the signals received from target cells that activate the mobilization of secretory lysosome granules [48]. This result also suggests a prominent role for CD40 signaling in FasL induction in B cells, as even the small population of B cells that survived with only CD40L stimulation expressed FasL and had measurable FasL-mediated killer function. Similar effects of CD40 ligation on FasL expression have been noted in mouse Langerhan’s cells, human hepatocytes and ovarian cancer cells [49]–[51]. Additionally, CD40 signaling results in down-regulation of BCL6, a transcription factor that competes with STAT3 for DNA binding sites [49], [50]. CD40 signaling therefore enhances STAT3 binding and facilitates the downstream effects of cytokines that signal through STAT3 by reducing levels of BCL6. The IL-5 receptor has been shown to activate STAT5, a transcription factor which shares many binding sites with STAT3 throughout the genome [48]. Therefore, it is possible that CD40 signaling acts in synergy with IL-5-receptor signaling to induce a killer phenotype in B cells. Using a B cell-derived cell line, we found that both CD40L and IL-5 stimulation individually induced FasL expression in an additive manner, suggesting that these two pathways might work in concert to increase FasL expression in B cells (Figure S3).

While expression of IL-4 and IL-5 is coordinately regulated in Th2 cells, other cell types secrete IL-5 and little or no IL-4. Among these cells are the recently-identified populations of innate lymphoid cells [52]–[54]. These cells are the major producers of IL-5 under homeostatic conditions, and while present but infrequent in the spleen, they are heavily enriched in locations where immunoregulation is essential for maintaining tolerance to innocuous environmental antigens and commensal microbes, such as lymphoid structures associated with the lung and intestine. One role for these innate IL-5-producing lymphoid cells might therefore be to help preserve immune homeostasis at these sites through IL-5-mediated induction of potentially regulatory B cell functions. It has also been reported that as murine Th2 cells are activated over time they transition from producing both IL-4 and IL-5 to producing exclusively IL-5 [55]. This is consistent with our previous finding that mice chronically infected with *S. mansoni* have increased numbers of FasL^+^ B cells [16], and suggests a model in which the return to homeostasis at the cessation of type 2 responses includes IL-5-mediated induction of killer B cells.

It has been suggested that, rather than existing as a homogenous population, B cells with immunosuppressive functions can arise from both B-1 and B-2 cells [56]. In this case, it is likely that different growth factors would differentially act on B cells from these different lineages. Recently the cytokine IL-21 was identified as a growth factor for IL-10-producing B cells using a similar culture system as we have used in this study [57]. As IL-21 is associated with the growth of follicular B cells, it is possible that IL-21 drives the growth of B-2-derived regulatory B cells, while IL-5 induces the growth of B-1-derived regulatory B cells. Given their differing niches, regulatory B cells derived from B-2 cells might be more effective in inhibiting autoimmunity in the peripheral lymphoid organs, whereas B-1-derived regulatory cells might be more effective at mucosal sites. More work is required, however, to clarify the differences and similarities of B cells stimulated with IL-21 or IL-5, as these two types of B cells might also target different cells *in vivo*.

Since B cells stimulated with IL-5 and CD40L secrete IL-10 and are potent inducers of apoptosis in activated T cells, we have made several attempts to assess their immunosuppressive function *in vivo* by adoptive transfer in a collagen-induced arthritis model. Our results thus far have failed to clearly define an effect for these transferred cells, as mice receiving untreated B cells or PBS have a similar disease course as those receiving IL-5-stimulated B cells (data not shown). B cells stimulated with IL-5 and CD40L, although enriched for B cells with killer function, still display heterogeneity. Given the reported pleiotropic effects of IL-5 on B cells outlined above, it is possible that IL-5 and CD40L stimulation results in expansion of a population of cells that is enriched for B cells with potential regulatory functions but not exclusively immunosuppressive in nature. Recently-published data reporting the strong effects of IL-21 on regulatory B cell growth showed a similar outcome, as *in vitro* treatment of CD19^+^ B cells with IL-21 led to enrichment of IL-10-producing B cells, but IL-10-producing B cells still comprised only a minority of B cells in culture [57]. It is also possible that, as mentioned above, B-1-derived regulatory B cells exert their immunosuppressive effects at locations distinct from those of B-2-derived regulatory B cells, and therefore the function of these cells would be better assessed in a different model system. Further work is therefore warranted to definitively demonstrate a role for IL-5 in the generation of immunosuppressive B cells *in vivo*, and this is an important caveat to our study.

In summary, the data presented herein characterize the phenotype of FasL^+^ B cells and demonstrate that FasL^+^ B cells are enriched in the CD5^+^CD1d^high^ B cells subset. A portion of FasL^+^ B cells express IL-10 in addition to FasL, but these FasL^+^IL-10^+^ B cells are extremely rare in naïve mice, representing <0.1% of all splenic B cells. Additionally, we show here for the first time that stimulation with IL-5 and CD40L expanded a population of B cells with potent FasL-mediated T cell-killing activity, and IL-5 enhanced IL-10 secretion from CD40L-stimulated B cells. Finally, IL-4 strongly inhibited the B cell killing activity mediated by IL-5, suggesting that cross-talk between these type-2 cytokines might be important in modulating the activity of killer B cells. Understanding the specifics of this IL-4/IL-5 axis in regards to B cell function could provide new targets for the design of therapeutic strategies for patients with autoimmune disorders or other inflammatory conditions.

## Materials and Methods

### Ethics Statement

All protocols involving animal subjects were approved by the University of Michigan Committee on the Use and Care of Animals.

### Mice

Wild-type DBA/1LacJ mice were obtained from our breeding colony at the University of Michigan or purchased from Jackson Laboratories. The cII-TCR transgenic mice were generated on the DBA/1LacJ background and recognize an immunodominant epitope of type 2 collagen (cII_259-272_) when presented by the MHC class II I-A^q^ molecule expressed in DBA/1 mice [18]. The presence of the transgene was confirmed by polymerase chain reaction genotyping in all animals.

### Flow Cytometry

Fluorochrome-conjugated antibodies and isotype controls were obtained from BD Biosciences (anti-CD19-APC, anti-CD19-PECy7, anti-B220-PECy7, anti-CD93-FITC, anti-CD23-FITC, anti-IgM-PECy7, anti-CD21-PE, anti-CD24-PE, anti-CD5-APC, anti-CD1d-FITC, anti-CD125-PE, anti-FasL-PE, anti-FasL-Biotin) or Biolegend (anti-CD3-APC, anti-CD9-FITC). Cells were incubated with anti-CD16/CD32 Fc Block (BD Biosciences) prior to staining and analyzed on an Accuri C6 flow cytometer. In all analyses, propidium iodide was used to exclude dead cells and doublets were excluded by gating based on forward scatter height and area channels. Data were analyzed using Cytobank web-based software [58] or FlowJo v7.6.5 (Tree Star, Inc.).

### Fluorescence-Activated Cell Sorting

Splenocytes from naïve mice were stained with anti-CD19-APC and anti-FasL-PE (BD Biosciences). CD19^+^FasL^−^ and CD19^+^FasL^+^ populations were then isolated using a FACSAria II (BD Biosciences).

### Identifying IL-10-producing B Cells

FasL^−^ and FasL^+^ B cell populations obtained by FACS were cultured for five hours with 50 ng/mL phorbol myristate acetate (PMA), 1 µg/mL ionomycin, and 5 µg/mL lipopolysaccharide (LPS) in the presence of monensin (GolgiStop, BD Biosciences). B cells were then fixed (4% paraformaldehyde) and permeabilized (0.5% Saponin, 0.2% BSA, 0.1% sodium azide in phosphate buffered saline) prior to staining with anti-IL10-FITC or isotype control antibody (BD Biosciences). No cells staining positive for IL-10 were detected in cultures without stimulation or in B cells treated with monensin in the absence of PMA/ionomycin/LPS stimulation (data not shown).

### B Cell Isolation and Culture

B lymphocytes were positively-selected from single-cell splenocyte suspensions using anti-CD19-coated magnetic beads (Miltenyi Biotec). Isolated B cell populations were routinely >95% pure. Mouse CD40L-transduced NIH-3T3 fibroblasts (a gift from Dr. Kevin McDonagh) were maintained in Dulbecco’s modified Eagle’s medium (DMEM) supplemented with 10% calf serum, penicillin/streptomycin, and L-glutamine [59]. For B cell stimulation cultures, CD19^+^ cells were added to a confluent layer of irradiated (30 Gy) CD40L-expressing fibroblasts in 24- or 96-well flat-bottomed culture plates in the presence or absence of 50 ng/mL recombinant mouse IL-5 and/or IL-4 (Peprotech Inc.). All stimulations were performed in DMEM supplemented with 10% fetal calf serum, penicillin/streptomycin, L-glutamine, 50 µg/mL transferrin, and 5 µg/mL insulin. After 5 days in culture, supernatants and B cells were collected for further study. Viable cells were enumerated using trypan blue exclusion.

### Proliferation Assay

B cells were cultured with irradiated CD40L-expressing fibroblasts in the presence or absence of IL-5. After 4 days, ^3^H-thymidine was added (5 µCi final concentration) and cells were harvested after 18 hours in culture. Radioactivity was assessed by a scintillation counter. Control wells containing CD40L-fibroblasts alone or B cells cultured on non-transduced NIH-3T3 fibroblasts showed no detectable proliferation in response to IL-5 (data not shown).

### Immunoblotting

Cell lysates from equal numbers of freshly-isolated CD19^+^ cells and B cells stimulated for five days with CD40L-expressing fibroblasts and cytokines were separated by SDS-PAGE and transferred to a PVDF membrane. Membranes were blocked and incubated with polyclonal rabbit anti-FasL IgG (Millipore) or anti-β-Actin (Cell Signaling). Antibody binding was detected with an anti-rabbit IgG-HRP secondary antibody (Cell Signaling) and ECL reagent (Thermo Scientific).

### Apoptosis Assay

Splenocytes were harvested from cII-TCR transgenic mice and stimulated with 2 µg/mL concanavalin A for three days. CD4^+^ T cells were then negatively selected by magnetic activated cell sorting (MACS) and cultured with freshly-isolated CD19^+^ B cells or B cells stimulated for 5 days on CD40L-expressing fibroblasts with or without IL-5. CD4^+^ T cell populations were >90% CD4^+^ after negative selection. Apoptosis assays were performed in RPMI supplemented with 10% fetal calf serum, penicillin/streptomycin, L-glutamine, HEPES, sodium pyruvate, and β-mercaptoethanol. After 18 hours of co-culture (1∶1 effector-to-target ratio), cells were collected and stained with anti-CD4-PE, Annexin V-FITC (BD Biosciences), and propidium iodide. Prior to co-culture with CD4^+^ T cells, some B cells were incubated with 10 µg/mL anti-FasL blocking antibody (R&D Systems) or an isotype control antibody for one hour and washed. To assess FasL-mediated “fratricide” between CD4^+^ T cells, control wells with T cells alone contained these antibodies for the full duration of culture. Apoptosis was measured by flow cytometry as the frequency of Annexin V^+^ cells within the CD4^+^ gated population.

### Enzyme-linked Immunosorbent Assay (ELISA)

B cells were cultured on CD40L-expressing fibroblasts for five days in the presence or absence of IL-5. Equal concentrations(7.5×10^5^ cells/mL) of stimulated B cells and freshly-isolated CD19^+^ B cells were then cultured in 96-well flat-bottomed plates with either no further stimulation, or with PMA (50 ng/mL) and ionomycin (1 µg/mL). Supernatants were collected after 24 hours, and IL-10 was quantified by ELISA following manufacturer’s protocols (R&D Systems).

### Statistics

The student’s *t* test was used to compare experimental groups using GraphPad Prism 5.0 software. Levels of significance are denoted throughout as follows: *p<0.05, **p<0.01, ***p<0.001, and ****p<0.0001. All graphs depict the mean ± SEM of replicate conditions/animals.

## Supporting Information

Figure S1 CD23^+^CD21^high^ B cells are not enriched among FasL^+^ B cells. (**A**) FasL^−^ and FasL^+^ B cells were stained for co-expression of CD23 and CD21. (**B**) The frequency of B cell populations as gated in (A) among FasL^−^ and FasL^+^ B cells in replicate animals was measured. (mean ± SEM)
(TIF)

Figure S2 Staining protocols for identifying IL-10-expressing B cells by intracellular staining eliminate FasL staining. Short-term culture and fixation reduce FasL surface staining in B cells. (**A**) Splenocytes from naïve mice were cultured for 6 hours with PMA (50 ng/mL), ionomycin (1 µg/mL) and LPS (5 µg/mL), stained with anti-CD19 and anti-FasL (or isotype control), then fixed and stained for intracellular IL-10. CD19^+^ cells were assayed for surface expression of FasL and intracellular IL-10. (**B**) Surface expression of FasL was assessed on CD19^+^ cells immediately *ex vivo* and after 6 hrs in culture with no further stimulation. The first set of panels depicts surface staining on living cells, and the second set of panels depicts staining on cells after fixation as in (A).
(TIF)

Figure S3 CD40L and IL-5 have additive effects on FasL levels in a B cell-derived cell line. The murine B cell-derived hybridoma cell line CIIC1 was cultured for two days with CD40L-expressing fibroblasts, IL-5 (50 ng/mL), or both CD40L-expressing fibroblasts and IL-5. Cell lysates from each condition were then probed for FasL and β-Actin proteins by immunoblot as in Figure 5G of the main text. The CIIC1 hybridoma was generated by fusing the Ag8.653 myeloma cell line with a B cell from a DBA/1 mouse immunized with chick type-II collagen emulsified in complete Freund’s adjuvant.
(TIF)
