# Supplementary figures and images for: Interleukin-5 Supports the Expansion of Fas Ligand-Expressing Killer B Cells that Induce Antigen-Specific Apoptosis of CD4+ T Cells and Secrete Interleukin-10
Source: PLoS One. 2013 Aug 5;8(8):e70131. doi: 10.1371/journal.pone.0070131 (PMC3734024; doi:10.1371/journal.pone.0070131)

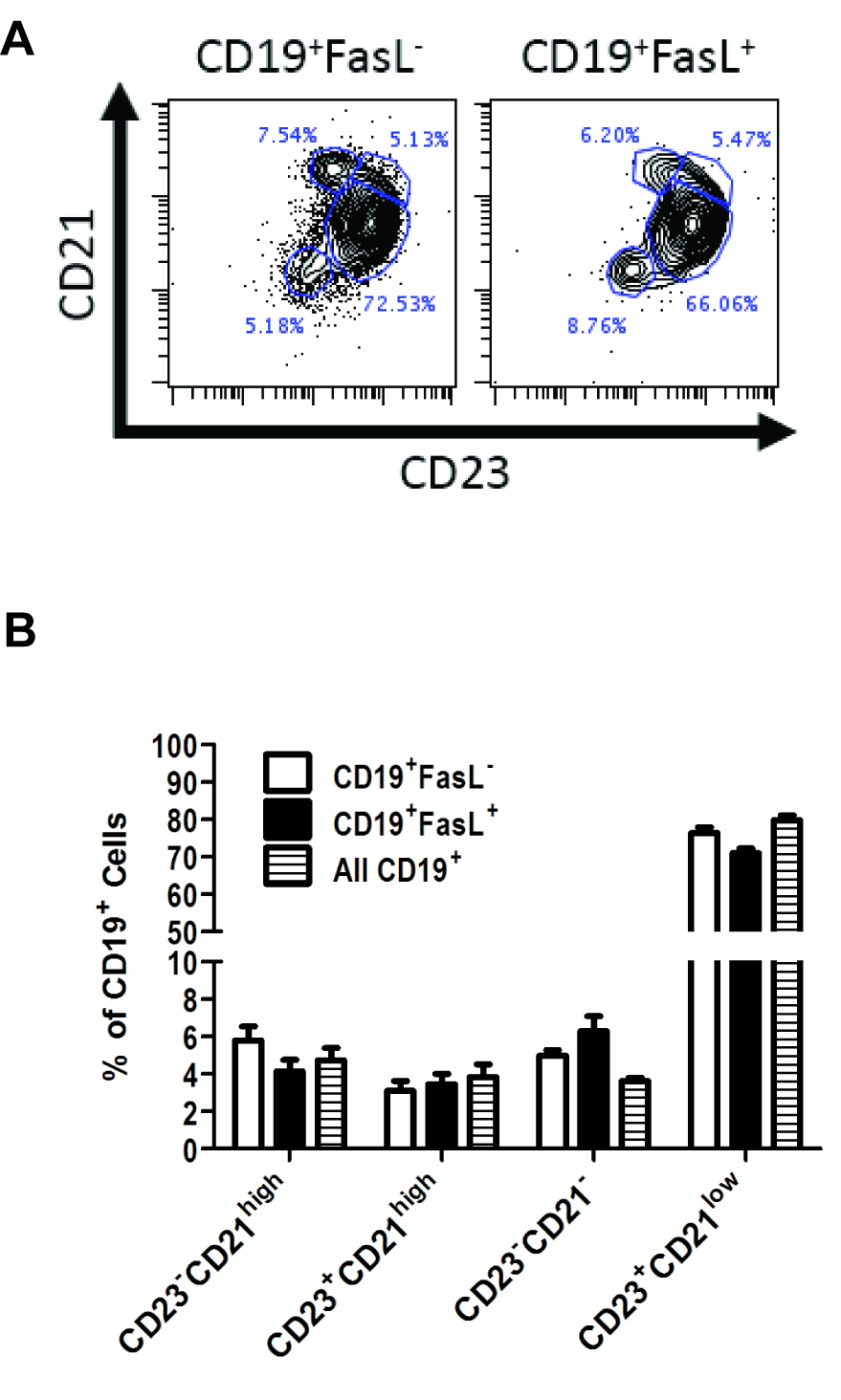

Supplement: Figure S1 — CD23+CD21high B cells are not enriched among FasL+ B cells. (A) FasL− and FasL+ B cells were stained for co-expression of CD23 and CD21. (B) The frequency of B cell populations as gated in (A) among FasL− and FasL+ B cells in replicate animals was measured. (mean ± SEM) (TIF) [file pone.0070131.s001.tif]

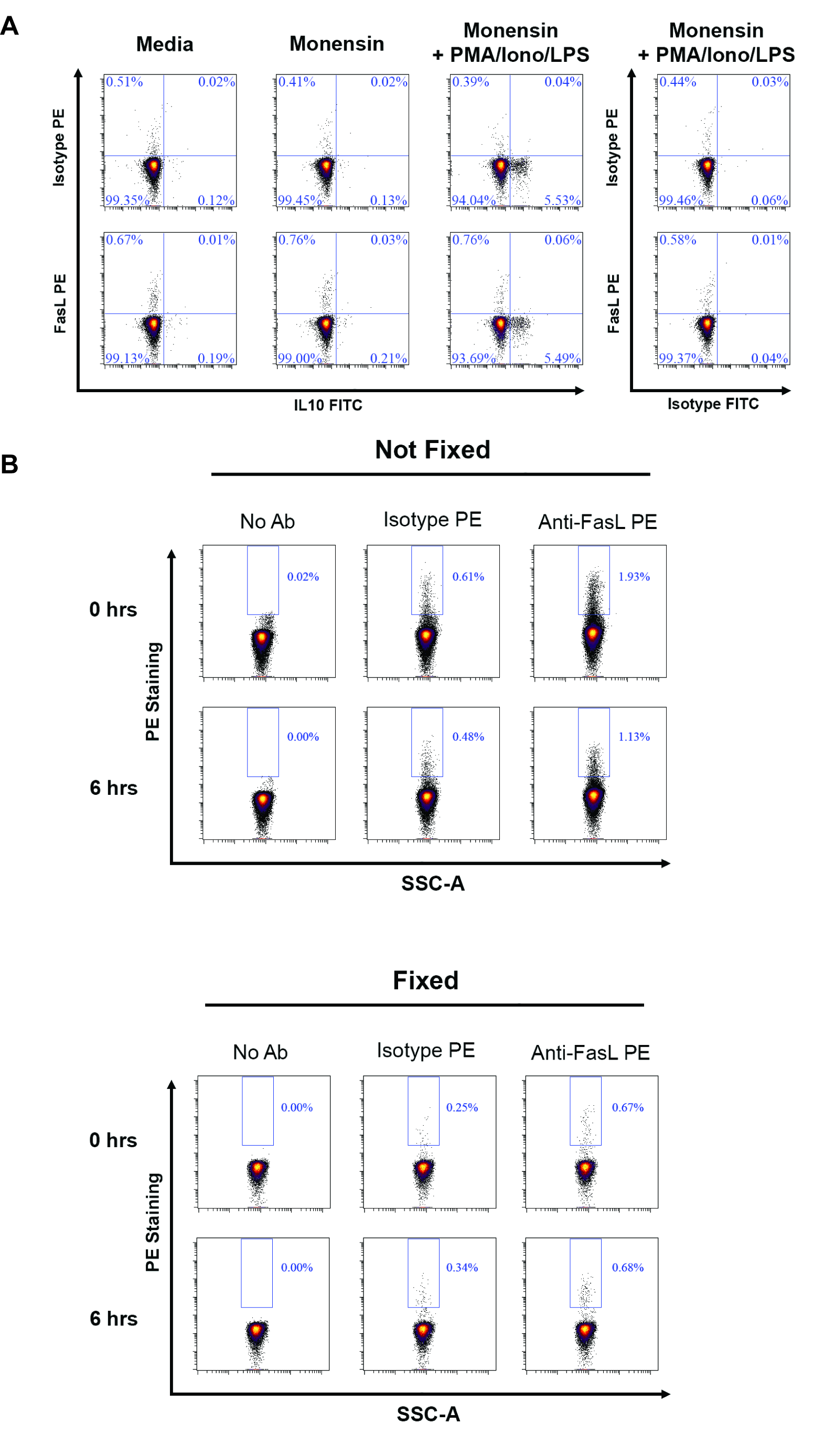

Supplement: Figure S2 — Staining protocols for identifying IL-10-expressing B cells by intracellular staining eliminate FasL staining. Short-term culture and fixation reduce FasL surface staining in B cells. (A) Splenocytes from naïve mice were cultured for 6 hours with PMA (50 ng/mL), ionomycin (1 µg/mL) and LPS (5 µg/mL), stained with anti-CD19 and anti-FasL (or isotype control), then fixed and stained for intracellular IL-10. CD19+ cells were assayed for surface expression of FasL and intracellular IL-10. (B) Surface expression of FasL was assessed on CD19+ cells immediately ex vivo and after 6 hrs in culture with no further stimulation. The first set of panels depicts surface staining on living cells, and the second set of panels depicts staining on cells after fixation as in (A). (TIF) [file pone.0070131.s002.tif]

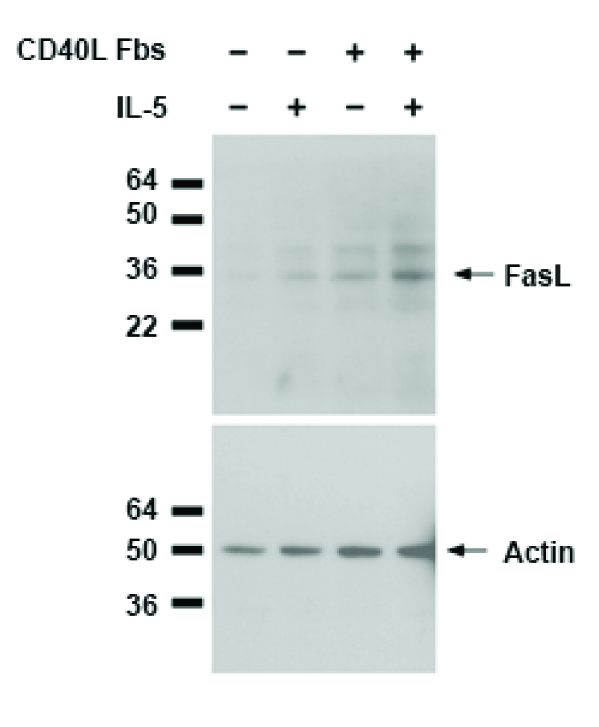

Supplement: Figure S3 — CD40L and IL-5 have additive effects on FasL levels in a B cell-derived cell line. The murine B cell-derived hybridoma cell line CIIC1 was cultured for two days with CD40L-expressing fibroblasts, IL-5 (50 ng/mL), or both CD40L-expressing fibroblasts and IL-5. Cell lysates from each condition were then probed for FasL and β-Actin proteins by immunoblot as in Figure 5G of the main text. The CIIC1 hybridoma was generated by fusing the Ag8.653 myeloma cell line with a B cell from a DBA/1 mouse immunized with chick type-II collagen emulsified in complete Freund’s adjuvant. (TIF) [file pone.0070131.s003.tif]
